# Cell Death Control: The Interplay of Apoptosis and Autophagy in the Pathogenicity of *Sclerotinia sclerotiorum*

**DOI:** 10.1371/journal.ppat.1003287

**Published:** 2013-04-11

**Authors:** Mehdi Kabbage, Brett Williams, Martin B. Dickman

**Affiliations:** 1 Department of Plant Pathology, University of Wisconsin-Madison, Madison, Wisconsin, United States of America; 2 Centre for Tropical Crops and Biocommodities, Queensland University of Technology, Brisbane, Queensland, Australia; 3 Institute for Plant Genomics and Biotechnology, Texas A&M University, College Station, Texas, United States of America; 4 Department of Plant Pathology and Microbiology, Texas A&M University, College Station, Texas, United States of America; 5 Center for Cell Death and Differentiation, Texas A&M University, College Station, Texas, United States of America; Oregon State University, United States of America

## Abstract

Programmed cell death is characterized by a cascade of tightly controlled events that culminate in the orchestrated death of the cell. In multicellular organisms autophagy and apoptosis are recognized as two principal means by which these genetically determined cell deaths occur. During plant-microbe interactions cell death programs can mediate both resistant and susceptible events. Via oxalic acid (OA), the necrotrophic phytopathogen *Sclerotinia sclerotiorum* hijacks host pathways and induces cell death in host plant tissue resulting in hallmark apoptotic features in a time and dose dependent manner. OA-deficient mutants are non-pathogenic and trigger a restricted cell death phenotype in the host that unexpectedly exhibits markers associated with the plant hypersensitive response including callose deposition and a pronounced oxidative burst, suggesting the plant can recognize and in this case respond, defensively. The details of this plant directed restrictive cell death associated with OA deficient mutants is the focus of this work. Using a combination of electron and fluorescence microscopy, chemical effectors and reverse genetics, we show that this restricted cell death is autophagic. Inhibition of autophagy rescued the non-pathogenic mutant phenotype. These findings indicate that autophagy is a defense response in this necrotrophic fungus/plant interaction and suggest a novel function associated with OA; namely, the suppression of autophagy. These data suggest that not all cell deaths are equivalent, and though programmed cell death occurs in both situations, the outcome is predicated on who is in control of the cell death machinery. Based on our data, we suggest that it is not cell death per se that dictates the outcome of certain plant-microbe interactions, but the manner by which cell death occurs that is crucial.

## Author Summary

During plant-microbe interactions, regulated cell death known as programmed cell death (PCD) can mediate both resistant and susceptible interactions. Sclerotinia sclerotiorum induces an apoptotic spreading cell death during infection, via the secreted virulence determinant oxalic acid. Oxalic acid deficient fungal mutants are non-pathogenic, and trigger a restricted cell death phenotype in the host that unexpectedly exhibits markers associated with the plant hypersensitive response. Using electron and fluorescence microscopy, chemical effectors and reverse genetics, we show that this restricted cell death is autophagic. Inhibition of autophagy rescued the non-pathogenic mutant phenotype. These findings indicate that autophagy is a defense response in this interaction and suggest a novel function associated with oxalic acid: the suppression of autophagy. Thus, the control of cell death, dictated by the plant (autophagy) or the fungus (apoptosis), can lead to opposing outcomes. We propose that the type of cell death is decisive to the outcome of certain plant-microbe interactions.

## Introduction

All higher organisms have developed an intrinsic program for cell suicide. These programs sense and monitor multiple internal and external cues and when deemed appropriate, instructs the cell to eliminate itself from the organism for the overall survival of the animal, plant or microbe [1]. Cell death programs span a continuum ranging from the highly ordered and tightly regulated apoptotic and autophagic cell deaths on one end of the spectrum, to necrosis on the other end. In a manner analogous to what occurs in mammals, worms and flies, programmed cell death regimes occur in plants as part of normal growth and development. Regulation of cell death pathways also occurs in response to abiotic and biotic stimuli [2]. Of particular note is the observation that during plant-microbe interactions, cell death programs can mediate both resistant and susceptible interactions [3]–[5]. Thus the control of cell death is vitally important for determining the outcome of an attack by a microbial invader. In multicellular organisms, apoptosis and autophagy have received the bulk of experimental attention and are recognized as principal means by which mammalian PCD occurs.

Apoptosis refers to a constellation of characteristic changes leading directly to cell death. Apoptosis is characterized by a cascade of tightly controlled checks and balances that must be successfully negotiated prior to reaching a point-of-no-return life/death decision that results in the irreversible execution of the cell. The implementation of this endogenous death program is often associated with several characteristic biochemical and morphological features including cell shrinkage, membrane blebbing, nuclear and chromatin condensation, externalization of phosphatidylserine and DNA fragmentation [2]. Although aspects of apoptotic cell death are conserved, the extent of these similarities to plant programmed cell death is not entirely clear, based on the available evidence it is reasonable to suggest that the conceptual and operational framework for PCD is conserved in a transkingdom manner.

Autophagy (self eating) is a major catabolic process in which proteins and damaged organelles are engulfed and sequestered in characteristic double membrane vesicles termed autophagosomes. This cellular cargo is delivered to lysosomes (mammals) or vacuoles (plants) for degradation and recycling [6]. Autophagy is a complex process requiring a large number of genes initially identified during starvation in yeast (“atg” genes), many of which have mammalian and plant homologs. Under conditions of environmental, nutritional or metabolic duress, this pathway provides degradation products of cellular reserves generating nutritional building blocks, thus maintaining energy homeostasis in a potentially lethal situation [7]. Autophagy has been implicated in an increasing range of environmental conditions, physiological stresses, developmental transitions and more recently pathological states [8]. A growing body of evidence suggests that autophagy plays a dual role both in cell survival and cell death; whether autophagy directly kills the cell is not clear, but it is believed that under conditions of severe stress, autophagic cell digestion increases the chances of cell survival. Thus autophagy can be viewed as a survival strategy to avoid (extensive) cell death by promoting cell death.

In contrast to mammalian cells, relatively less is known about PCD specifics in plants, although several physiological processes in plants are apoptotic-and/or autophagic-like. The *Arabidopsis thaliana* genome harbors more than 20 genes which display high homology to the established yeast *ATG* genes, but in some cases, functional conservation awaits demonstration [9]. As in mammals, plant autophagy pathways have been functionally implicated in the recycling of nutrients during starvation, and several abiotic stresses [9], [10]. Emerging data suggest that autophagy pathways are also involved in the regulation of the plant hypersensitive response (HR) and basal immunity, thus linking autophagy with plant defense [4].

*Sclerotinia sclerotiorum* is a cosmopolitan necrotrophic fungal pathogen with a broad host range (>400 plants species). A key factor governing the pathogenic success of this fungus is the secretion of the non-selective phytotoxin, oxalic acid (OA) [11]. *S. sclerotiorum* mutants defective in OA production are non-pathogenic. Oxalic acid can contribute to pathogenesis in a number of ways (e.g. acidification, chelation of Ca^2+^, low pH activation of degradative enzymes etc.) that augment fungal colonization of host plants [12]. Recently, additional novel roles for OA during *S. sclerotiorum* pathogenicity have been identified. We have suggested and provided evidence for the ability of OA to manipulate signaling pathways involved in the generation of reactive oxygen species (ROS) in the host [13], [14]. OA is also a fungal effector that induces apoptotic cell death with hallmark features including DNA laddering, TUNEL reactive cells and chromatin condensation [5]. Recently we demonstrated that OA-deficient mutants are not only non-pathogenic but curiously trigger markers and phenotypes reminiscent of a hypersensitive (HR) response including callose deposition, elicitation of a strong oxidative burst, induction of phenolic compounds and restricted, clearly delimited growth of the fungus [14]. In contrast, wild type *S. sclerotiorum* secreted OA suppresses all of these responses, and of particular note, is the complete absence of the host oxidative burst during the early stages of fungal growth and disease development [14]. Using a redox GFP reporter expressed in *Nicotiana benthamiana*, we showed an early induction of reductive stress in host cells in advance of the growing fungus as a result of oxalate secretion. We have suggested that this induced reductive state facilitates fungal dampening of the host oxidative burst, consistent with our previous reports [13]. We have also suggested that this inhibition of ROS affords the fungus valuable time for unimpeded establishment of infection. Once infection is established, *S. sclerotiorum* transitions to a necrotroph life style and induces the generation of ROS, triggering apoptotic PCD and a rapid spread of cell death and disease [14].

Here we report a significant contrast in this host-fungal interaction. As noted, wild type “compatible” *S. sclerotiorum* induces an apoptotic-like cell death in the host. We have shown that inoculation of wild type *S. sclerotiorum* to transgenic *Arabidopsis* carrying the *C. elegans CED-9* anti-apoptotic gene resulted in a resistant phenotype [15]. Inoculation of *Arabidopsis* with an OA-deficient non-pathogenic mutant strain of *S. sclerotiorum* (A2) exhibited a restricted growth-cell death phenotype, accompanied by HR associated markers similarly observed in both the Col-0 and *CED-9* plants. This observation is important, because it not only implies that the plant can recognize and respond to the A2 mutant (as manifested by the “HR”), but also suggests that the cell death phenotypes triggered by *S. sclerotiorum* wild type and A2 may differ. Here we report the results of further studies based on these observations and provide evidence that these programmed cell deaths do indeed, differ. Whereas wild type *S. sclerotiorum* induces a runaway apoptotic cell death, we provide several lines of evidence (genetic, chemical, microscopic) indicating that autophagic cell death is occurring and is responsible for the restricted growth phenotype associated with the A2 mutant. Wild type *S. sclerotiorum* or oxalate alone, suppresses this autophagic response. These findings indicate that distinct host cell death responses occur during wild type and mutant *S. sclerotiorum* challenges and provide evidence for the cytoprotective and defensive roles of autophagic cell death during plant-necrotrophic pathogen interactions. The two types of cell death observed are either host induced (autophagy) or are pathogen controlled (apoptosis) with opposing outcomes. These data suggest that not all cell deaths are equivalent, and though programmed cell death occurs in both situations, the outcome is predicated on who is in control of the cell death machinery and the type of cell death. These responses reflect the life-style of the pathogen and the genetic background of the host; in both interactions host cell death is triggered and central for a given response but with very different consequences.

## Results

### Cell death phenotypes differ between wild type and mutant *S. sclerotiorum*

**Figure 1:**
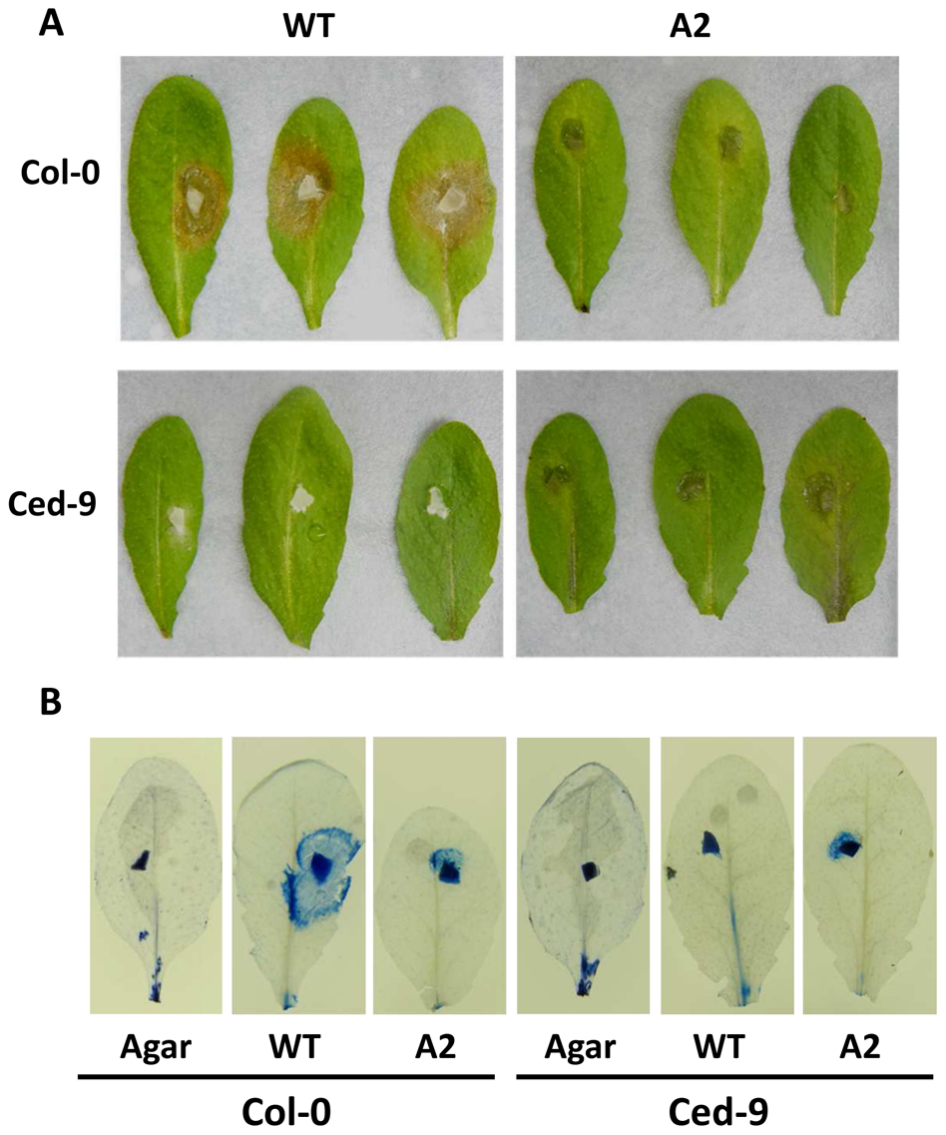
Expression of *ced-9* in *Arabidopsis* inhibits wild type infection but does not affect the A2 phenotype. Agar plugs containing actively growing cultures of wild type *S. sclerotiorum* (strain 1980) and the OA deficient A2 mutant were inoculated onto Col-0 and *ced-9* expressing *Arabidopsis* leaves. (A) Wild type inoculations onto Col-0 plants resulted in typical lesions for this pathogen including a rapid, spreading cell death; however, infection was completely suppressed in *ced-9* expressing plants. The expression of this gene had no effect on the A2 phenotype. (B) Trypan blue staining indicates the extent of cell death for each genotype/strain combination, including an agar plug control. All images were recorded 48 hours post inoculation.

Previously, we showed that transgenic plants expressing anti-apoptotic Bcl-2 family members inhibited wild type *S. sclerotiorum* induced programmed cell death and disease development. Given the phenotype of the A2 mutant, we were interested in comparing phenotypes in transgenic plants expressing anti-apoptotic genes following fungal challenge. Transgenic *Arabidopsis* Col-0 plants carrying *CED-9* (GeneID:3565776), the *C. elegans* homolog of human *BCL-2*, were inoculated with agar plugs containing wild type *S. sclerotiorum* and A2. As expected, growth of wild type *S. sclerotiorum* was markedly inhibited in *CED-9* leaves compared to Col-0 control plants (Fig. 1A). This fungus induces hallmark apoptotic features, such as TUNEL positive nuclei and DNA laddering in the host [5]. However, the A2 phenotype remained unchanged when inoculated onto wild type or transgenic plants expressing *CED-9* (Fig. 1A). In some cases, necrosis was observed along the central vein of *CED-9* leaves inoculated with the A2 strain (Fig. 1A), however, such necrosis was restricted in spread and was not always present. Trypan Blue staining was used to evaluate the extent of cell death at the inoculation point in these tissues and showed no differences in the extent of plant tissue death between *CED-9* transgenics and Col-0 plants inoculated with A2 (Fig. 1B). This indicates that *CED-9* was unable to suppress the cell death induced by A2 and further suggests that the mechanism underlying cell death differs from the *S. sclerotiorum* wild type strain. These observations also suggest that wild type *S. sclerotiorum* evades/suppresses plant defense responses as opposed to the prevailing view that this fungus simply overwhelms the defenseless host with its battery of degradative enzymes and toxic molecules. In the case of the A2 mutant, the plant appears to recognize the fungus (and/or the fungus is unable to suppress this recognition) and intriguingly, a HR-like response ensues. This is of note as necrotrophs epitomized by *S. sclerotiorum* have not been associated with these types of host responses. Moreover, in both interactions, cell death occurs but clearly with different consequences.

**Figure 2:**
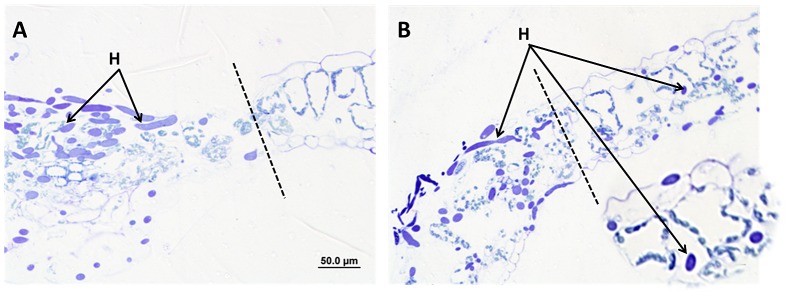
Microscopic examination of cross sections of tomato leaves at the leading edge of the lesion following fungal inoculation. *S. sclerotiorum* A2 (A) and wild type (B) strains were inoculated onto tomato leaves using colonized agar plugs. 24 hours post inoculation; leaves were post-fixed in osmium tetroxide, and embedded in Spurr's epoxy resin. A microtome was used to cut 400 nm sections. Toluidine blue stain was used to reveal fungal hyphae. H = hyphae. The dotted line represents the leading edge of the visible lesion. Images were collected using an Olympus DP 70 camera and processed with Olympus DP Controller software, version 2.2.1.227.

Since we observed a distinct line of demarcation where A2 mycelial growth was restricted, we microscopically examined tomato leaf cells following inoculation with both the A2 mutant and wild type fungus. Twenty-four hours after inoculation, leaves were fixed and embedded in Spurr's epoxy resin. 400 nm sections were cut and stained with Toluidine blue. Light microscopy showed that fungal mycelium was unable to progress beyond the restricted area in the A2 interaction (Fig. 2A). Furthermore, the cellular content in the restricted area and immediately beyond the lesion edge (intact epidermal cells) was disorganized and organelles such as chloroplasts were no longer structurally distinct (Fig. 2A). In contrast, in the compatible wild type *S. sclerotiorum* infection, fungal mycelia were present beyond the lesion edge in intact cells (Fig. 2B). The viability of these cells was further confirmed in onion epidermal cells using the life and death stain, trypan blue (Fig. S1). In the A2 interaction, we also noted pronounced constriction of tomato leaf tissue thickness at the lesion boundary (Fig. 2A); though the significance of this observation is not yet clear, this leaf constriction coincides with the arrest of fungal growth. Conversely, during wild type *S. sclerotiorum* challenge, a virtually seamless transition from diseased to healthy tissue was observed, leaf thickness remained unaltered and plant cells beyond the lesion edge appeared intact, though fungal growth was evident in living tissue. This is an important observation because it indicates that this fungus first has a biotrophic phase during pathogenesis where cell viability is specifically maintained prior to the transition to necrotrophy and initiation of host apoptotic cell death.

### Lysotracker and MDC labeled structures are present following A2 challenge

Autophagy pathways have been implicated in host defense responses and restriction of pathogen access to host tissue [4]. Since the wild type strain induces an apoptotic PCD, we investigated whether autophagy is pertinent to the defense response against A2. Wild type and A2 inoculated leaf samples were stained with LysoTracker, a dye which stains acidic organelles, including autophagosomes [4]. Leaves inoculated with A2 accumulated a large number of punctate structures after 24 h (Fig. S2). No staining was observed in leaves inoculated with wild type *S. sclerotiorum* at the same time point. The pattern of LysoTracker staining suggests autophagic activity is occurring specifically during the A2 challenge.

**Figure 3:**
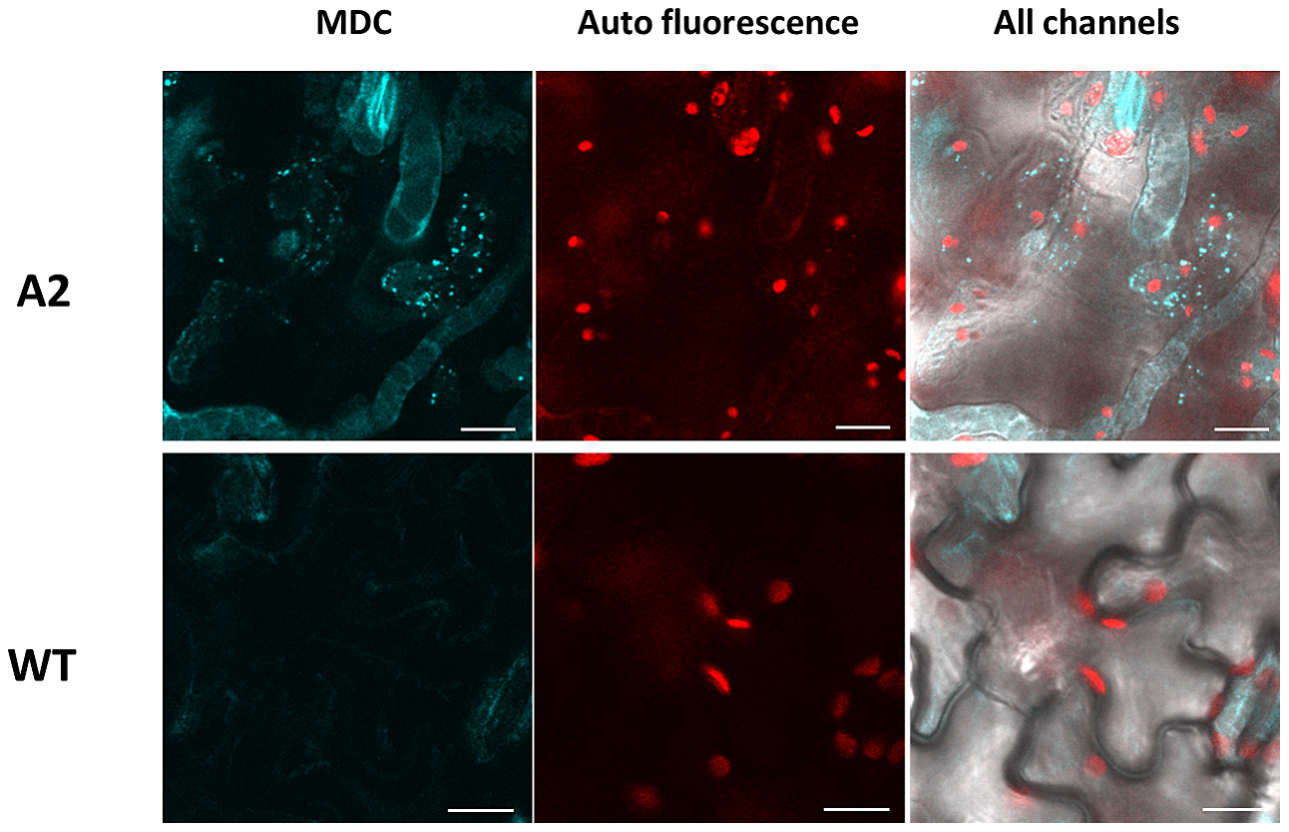
*S. sclerotiorum* A2 strain induces autophagic structures in plants. *S. sclerotiorum* wild type and A2 strains were inoculated onto tomato leaves using colonized agar plugs. 24 hours post inoculation; leaves were stained with 100 µM final concentration of MDC (Sigma) in PBS for 30 min. Fluorescence was visualized using an Olympus IX81 inverted fluorescence confocal microscope (Olympus systems, Germany), with an excitation wavelength of 335 nm and an emission wavelength of 508 nm. Images were collected using an Olympus DP 70 camera and processed with Olympus DP Controller software, version 2.2.1.227. Scale bar = 10 µm.

A limitation of lysotracker is specificity, since this stain binds all acidic organelles. To better confirm the presence of autophagy related structures during the A2 interaction with plant tissue, a more specific dye, Monodansylcadaverine (MDC) was used. MDC is an autofluorescent dye that specifically labels autophagosomes. Studies in mammalian cells have shown that MDC specificity is attributed to its ability to bind lipid molecules present in autophagosomes as well as ion trapping due to the acidic nature of these structures [16]. Contento et al., (2005) [17] demonstrated that MDC was also a suitable marker for autophagosome formation in plants. Both wild type and A2 strains were grown on Potato Dextrose Agar (PDA) and agar plugs containing actively growing mycelia were inoculated onto detached tomato leaves. After 24 h, leaves were incubated with 100 µM MDC solution for 30 min at 37°C and then washed with phosphate-buffered saline (PBS) to remove excess dye. Leaves were analyzed by fluorescence microscopy with excitation and emission wavelengths of 335 nm and 508 nm, respectively. As controls, non-inoculated detached leaves were also stained to rule out senescence as a source of fluorescence (Fig. S6). As shown in figure 3, 24 h after leaves were inoculated with A2, strong MDC staining of spherical structures was observed within the cells. These structures were mostly present within the cytoplasm and varied in size. In contrast, cells of leaves inoculated with the wild type strain were weakly stained at best, and did not exhibit MDC-stained vesicle-like structures as observed in plants inoculated with the A2 strain (Fig. 3). These results strongly suggest that A2 challenge induced the formation of MDC-labeled autophagosomal structures in plants and the induction of autophagy pathways in response to this fungal mutant strain.

### The *S. sclerotiorum* A2 mutant triggers the production of autophagosomes

**Figure 4:**
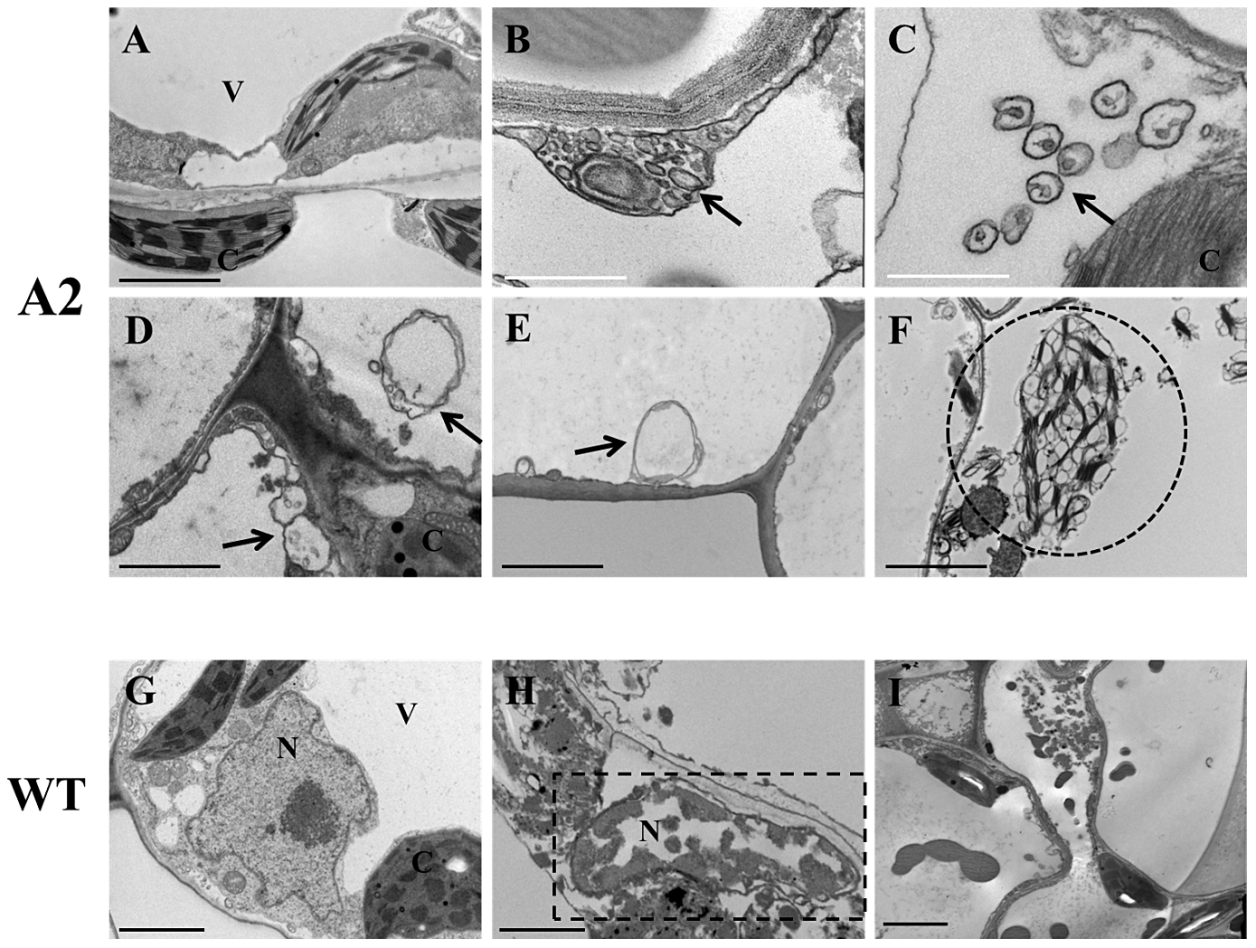
Transmission Electron Microscopy (TEM) fungal inoculated tomato leaves. Representative TEM images from four independent experiments. (A, G); Healthy non-inoculated leaf tissue. (B–F) Tomato leaves inoculated with the OA deficient A2 strain. (H,I) Tomato leaves inoculated with wild type *S. sclerotiorum*. Arrows, autolysosomal/autophagosomal-like structures; C, chloroplast; V, vacuole; N, nucleus; Circle, active dismantlement of chloroplast; Rectangle, chromatin condensation within the nucleus. Black scale bars = 2 µm, white scale bars = 1 µm. Sections were examined with a Phillips Morgagni 268 transmission electron microscope at an accelerating voltage of 80 kV. Digital images were recorded with a MegaViewIII digital camera operated with iTEM software.

Transmission Electron Microscopy (TEM) constitutes the most compelling evidence for the occurrence of autophagy. TEM was performed to more accurately determine whether A2 challenged plants trigger an autophagic mediated cell death response. Twenty-four hours after inoculation, tomato leaves were fixed in osmium, dehydrated and embedded in resin. Ultrathin sections were cut both within the confines of the lesion (no epidermal layer) and in the area immediately surrounding the lesion (intact epidermal layer). Our examination focused on ultrastructurally intact cells during the developmental sequence of events of the autophagy process prior to cellular demise. The TEM analysis provided unambiguous evidence for the presence of autophagy structural features following inoculation with the A2 mutant, but not in the wild type challenge (Fig. 4). Numerous small single membrane vesicles consistent with early lysosomal activity were observed in cells adjacent to the lesion (Fig. 4B), which were absent in control healthy tissue and wild type infected plants (Fig. 4A). Classic, diagnostic features associated with autophagic development were observed in A2 challenged tissue, including the presence of double membrane vacuoles with sequestered cellular content at various stages of degradation (Fig. 4C,D). Empty autophagosomes containing sequestered cargo that was completely digested were also observed (Fig. 4E). Similar ultrastructural features were observed by Rose et al (2006) [18] during starvation induced autophagy in *Arabidopsis* and in N mediated resistance to TMV by Liu et al (2005) [4]. In addition to degradation of cytoplasmic material as evidenced by the presence of autophagosomal structures, we also noted an active dismantling of chloroplasts (Fig. 4F). Chloroplast targeting “chlorophagy”, has been suggested to play a role in plant immune defenses [19]. In wild type infected tissue such structures were not observed (Fig. 4H&I) even in severely diseased tissue (Fig. 4I). However, chromatin condensation was visible in nuclei of cells of tissue infected with wild type *S. sclerotiorum* (Fig. 4H), in accordance with our previous observations that *S. sclerotiorum* plant challenge exhibit features associated with apoptotic programmed cell death [5]. Taken together, these data confirm the induction and presence of autophagosomes and autophagic responses during host plant interaction with A2. These data suggest that plant cells actively sense and recognize the A2 mutant mounting a defense response that includes autophagic cell death. Thus, apoptotic cell death induced by wild type *S. sclerotiorum* may suppress autophagy in plants; as has been observed in mammals where apoptosis has been shown to block autophagosome synthesis [20]. To test this possibility, host tissue was pre-treated with oxalic acid, an elicitor of apoptosis. OA pre-treatment prior to A2 challenge, significantly reduced the number of MDC stained vacuoles (Fig. S4). It is not clear whether OA directly suppress autophagy and apoptosis ensues, or the induction of apoptosis by OA overrides autophagy.

### Genetics and chemical analysis of autophagy

**Figure 5:**
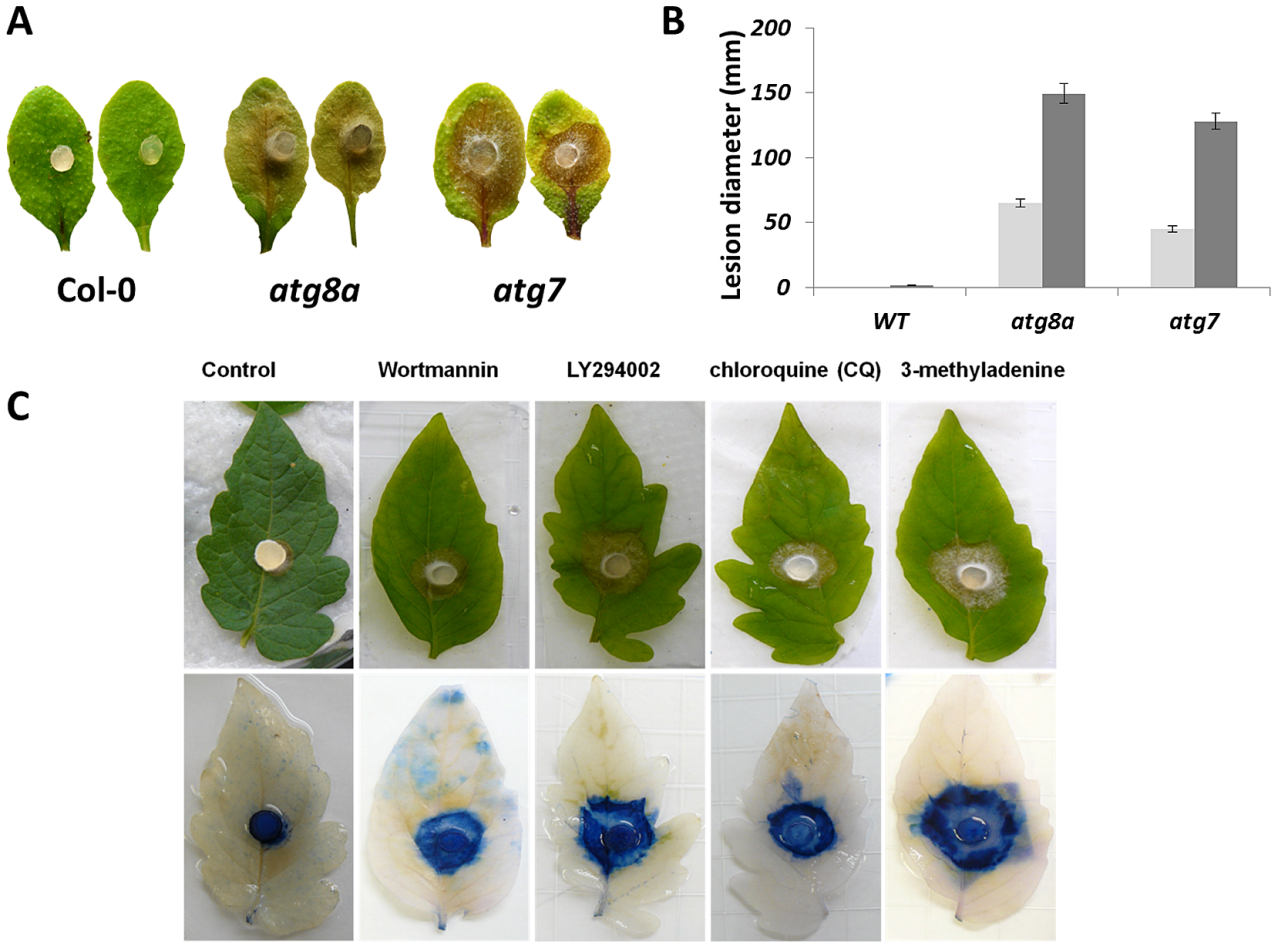
Inhibition of autophagy restores A2 pathogenicity. (A,B) Agar plugs containing actively growing cultures of the OA deficient A2 mutant were inoculated onto leaves of *Arabidopsis* Col-0 and select *Arabidopsis* autophagy mutant plants. These mutants showed enhanced susceptibility to the normally non-pathogenic A2 strain. Lesion diameter was monitored over time and all images were recorded 48 hours post inoculation. (C) Tomato leaves were either pre-infiltrated with water (control) or autophagy inhibitors Wortmannin, LY294002, Chloroquine (CQ), and 3-methyladenine (3-MA). Agar plugs containing actively growing A2 were placed on the infiltrated leaves to initiate infection. (B) 48 hours post inoculation; Trypan blue was used to determine the extent of fungal colonization and cleared with acetic acid and ethanol (1: 3, v/v). Images were taken 48 hours post inoculation.

If autophagy is responsible for defense against A2, then inhibition of autophagy should impact plant resistance. We therefore used a two-pronged strategy utilizing a genetics-based approach with several available autophagy defective *Arabidopsis* T-DNA insertion lines in combination with a chemical approach that exploited compounds known to inhibit the autophagic process. Four *Arabidopsis* autophagy-deficient knockout mutants of two genes (*atg7* and *atg8*) were acquired from the Arabidopsis stock center; two independent T-DNA knockout lines were used for each genotype to insure gene specific responses. These genes are orthologs of yeast genes and linked to autophagosome formation and nutrient deprivation [18]. *ATG7* has a role in the N-mediated cell death in response to TMV [4], [21]. Autophagy mutant and wild type leaves were inoculated with agar plugs containing mycelia from the A2 strain (Fig. 5A). Both mutant lines showed an enhanced susceptibility phenotype, albeit to varying levels depending on the particular mutation (Fig. 5A,B). Similar results were obtained with another OA-deficient strain of *S. sclerotiorum*, *Δsod1* (Fig. S3, [22]). The lesion size and rate of spread varied and was more pronounced in the *atg8* mutant lines. (Fig. 5A,B, Fig.S3). No differences were observed between the autophagy mutant lines and Col-0 plants when inoculated with the wild type *S. sclerotiorum* strain (Fig. S3). A2 inoculation of wild type plants resulted in the expected restricted lesion phenotype. These results suggest that interference with the autophagy machinery compromises resistance to A2 and is in accordance with a role for OA in the suppression of autophagy.

We next asked whether exogenous application of autophagy inhibitors could enhance OA mutant infection. We used 3-methyladenine (3-MA), Wortmannin, LY294002, and Chloroquine (CQ). 3-MA, Wortmannin, and LY294002 are specific inhibitors of phosphatidylinositol 3-kinase, a necessary component of the autophagy process [23]. Chloroquine has long been used in the treatment and prevention of malaria and inhibits the lysosomal acidification that is required for fusion between autophagosomes and lysosomes. Tomato leaves were pre-infiltrated with these inhibitors and inoculated with agar plugs containing A2. Challenged tomato leaves were more susceptible in all treatments, but to varying levels depending on the chemical with 3-MA and LY294002 pre-treatments being most permissive to A2 infection (Fig. 5C), the use of these chemicals had no effect on wild type *S. sclerotiorum* infection. Infiltration of healthy leaves ruled out direct toxicity of these chemicals as such leaves were unaffected in the time frame of the experiment (data not shown). Together, results indicate that the plant's ability to mount a defense response is compromised by several differentially targeted autophagic inhibitors consistent with the involvement and importance of autophagy as a key defense response against *S. sclerotiorum* A2.

### *Arabidopsis atg* mutants are unable to mount an oxidative burst in response to A2

**Figure 6:**
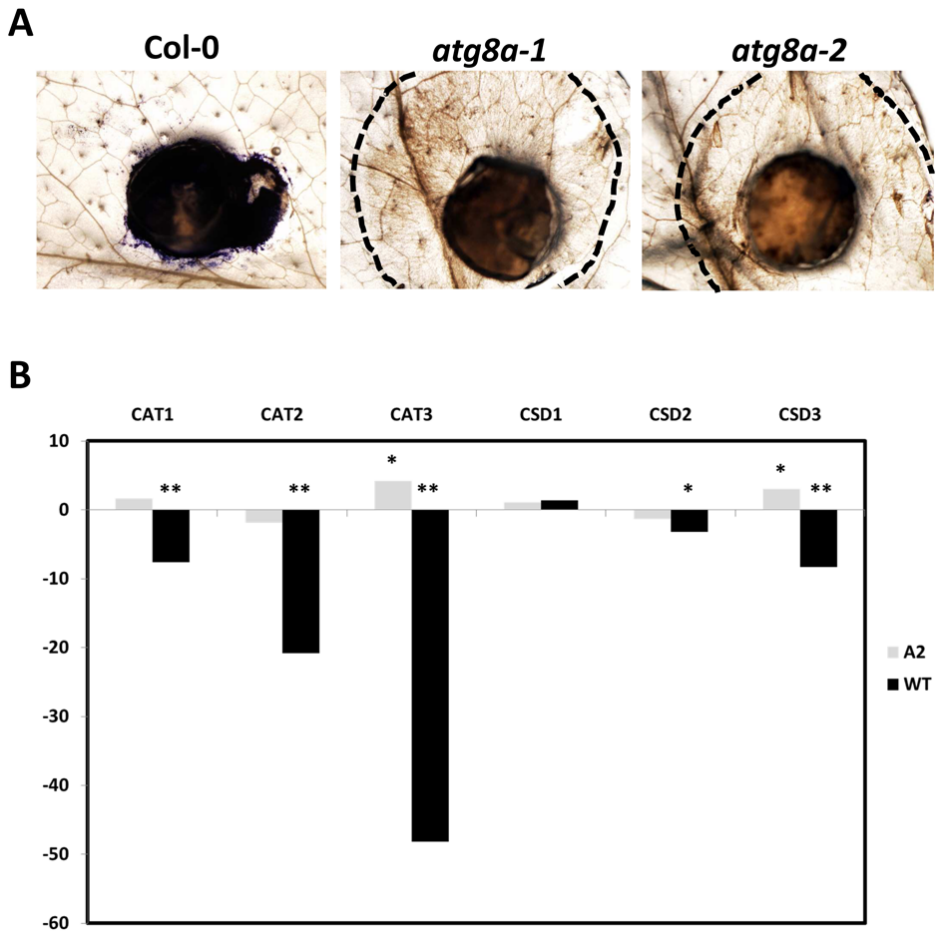
Oxidant accumulation in wild type and *atg* mutant plants. (A) NBT treated Arabidopsis (Col-0 and two independent *atg8a* mutant lines) following agar plug inoculation with the A2 mutant. Images were collected 48 hours post inoculation. Dotted lines represent the edge of the observable legion. (B) RT-PCR was used to evaluate the transcript levels of three catalases (CAT1, 2, and 3) and three superoxide dismutases in Col-0 plants following inoculation with wild type *S. sclerotiorum* (black bars) and the A2 mutant strain (grey bars). *>2 fold change, **>5 fold change.

Previously we showed that *S. sclerotiorum* secreted OA is a pathogenicity determinant and elicitor of plant programmed cell death [5], [11]. Furthermore, we demonstrated that in the absence of OA (i.e. OA mutants), the plant is now able to recognize this fungus, mount an oxidative burst, and halt disease progression [14]. Remarkably, ROS was virtually absent in leaf tissue challenged with wild type *S. sclerotiorum* [14]. In light of the sensitivity of the Arabidopsis *atg* knock-out mutants to the normally non-pathogenic OA deficient mutants, we determined the ROS status in these mutants in response to the A2. Wild type and *atg8a* Arabidopsis leaves were inoculated with the A2 mutant and stained with the superoxide indicator, nitroblue tetrazolium (NBT) 24 hours post-inoculation to detect superoxide. Consistent with our previous data, dark-blue formazan precipitates were observed in wild type plants following A2 inoculation (Fig. 6A), whereas the *atg8a* mutants had a significant reduction in superoxide accumulation and fungal hyphae were clearly visible within the confines of the lesion (Fig. 6A). The significant reduction in NBT staining indicates that superoxide production was impaired in *atg8a* mutants.

We also evaluated the expression profile of ROS related genes in *Arabidopsis* leaves in response to both the A2 and wild type *S. sclerotiorum* strains. The expression of several *Arabidopsis* catalases (CAT1, CAT2, and CAT3) and superoxide dismutases (CSD1, CSD2, and CSD3) were monitored by real-time PCR analysis. All catalase genes were markedly down regulated (>10 fold) in response to wild type *S. sclerotiorum*. In contrast CAT3 was significantly induced (>5 fold) in samples inoculated with the A2 mutant (Fig. 6B). No significant differences were observed in the expression of CAT1 and CAT2 in response to the A2 strain compared to the mock inoculated control. Though CSD1 levels remained unchanged, superoxide dismutase expression CSD2 (>5 fold) and CSD3 (>10 fold) were also significantly down regulated in samples inoculated with the wild type strain. Similar to CAT3, CSD3 (>5 fold) levels were significantly increased when the A2 strain was used. Consistent with ROS staining, the real-time data showed increases in *Arabidopsis* ROS gene expression, notably CAT3 and CSD3, in response to A2 inoculation and demonstrate an important role for ROS in the defense response against this mutant strain. This is consistent with the established connections between ROS and autophagy in diverse pathological conditions [24].

### Expression analysis of autophagy and defense related genes

Accumulation of several ATG protein-encoding transcripts has been reported in pathogen infected *Arabidopsis* plants [25]. Using quantitative PCR, we analyzed the expression pattern of several selected *Arabidopsis* autophagy genes in response to wild type and A2 fungal strains. The *ATG* genes with significantly different expression levels compared to control samples are shown in figure S5A. Consistent with the premise that autophagy plays a role in host defense against the A2 mutant; a range of previously characterized autophagy genes were significantly induced in leaf tissue inoculated with A2 but not in wild type infected leaves (Fig. S5A). Genes involved in the ATG8 conjugation pathway; *ATG8b*, *ATG8i* and *ATG18a* were induced in both wild type and A2 infections; however, the levels of expression of these genes were markedly higher upon A2 challenge and showed more than a 10 fold increase in expression as compared to wild type inoculated samples. *ATG8b* expression increased more than 100 fold in response to A2 (Fig. S5A). In the wild type inoculated samples, we observed that *ATG4*, *ATG8f*, and *ATG8g* were significantly down regulated. *ATG8g* expression in particular, was decreased by nearly 20 fold. This result is consistent with the suppression of autophagy by wild type *S. sclerotiorum*, via OA, and dampening of host defense responses. The overall expression pattern of autophagy related genes presented here suggests that autophagy is triggered in the plant host and is necessary for host defense against A2. The expression pattern of several defense related genes was also investigated, including the JA regulated gene *PDF1.2*; the induction of which has been associated with response to necrotrophic pathogens [26]. The salicylic acid markers PR1 and PR5, which are generally involved in defense against biotrophic pathogen were also evaluated. As shown in figure S5B, a dramatic increase (over 150 fold) in PDF1.2 levels was observed in response to the A2 mutant. PDF1.2 levels were also increased upon challenge with wild type *S. sclerotiorum*, however, these levels were considerably lower compared to those obtained following inoculation with the A2 strain. PR1 expression levels were similarly increased in both instances, suggesting that PR1/SA may not impact *S. sclerotiorum* pathogenicity. These findings suggest that elevated *ATG* gene expression in A2 inoculated *Arabidopsis* plants are in accordance with strong defense responses against this fungal strain.

## Discussion

In this study we demonstrate that the control of cell death governs the outcome of the *S. sclerotiorum*/plant interaction. These data also suggest this prototypical necrotroph has a biotrophic phase which occurs during the initial stages of disease establishment. Host defense responses, in particular the oxidative burst, are suppressed as the fungus grows through living tissue. Once the fungus is established, a transition to necrotrophy occurs and host cell death pathways are subverted inducing an apoptotic cell death. This fungal induced cell death provides nutrients that are exclusively for the benefit of the fungus. In non-pathogenic mutants, plant controlled cell death via autophagy is observed. If autophagy is blocked, genetically or pharmacologically, resistance is compromised and formerly non-pathogenic mutants are no longer restricted in growth. Thus pathogenic success occurs by fungal control of programmed cell death.

We examined the underlying mechanisms of PCD in fungal wild type and non-pathogenic mutant plant interactions. These studies were prompted by previous observations indicating wild type induced disease (spreading cell death) was inhibited by the expression of cytoprotective anti-apoptotic gene *CED-9* [15]; the oxalic acid mutant (restricted cell death phenotype), was unaffected by *CED-9* expression, suggesting a different cell death pathway might be operative. Given that the wild type fungus induces an apoptotic cell death, we hypothesized that an autophagy-like process might be important. There is no singular standard for assessing autophagic flux and activity. We utilized several non-overlapping methods to monitor and evaluate autophagic cell death activity including a combination of transmission electron microscopy, fluorescence microscopy, chemical and reverse genetics to further investigate the host response upon A2 mutant challenge. In particular, the TEM studies were informative, clearly showing morphological characteristics that are diagnostic for autophagy, including cargo containing double membrane autophagosomes and numerous membrane vesicles. Several independent *Arabidopsis* T-DNA insertional mutant lines defective in autophagy were permissive to A2 pathogenic development. A2 was able to grow and infect the *atg* mutant plant lines similar to what we observed in A2 inoculations with OA supplementation [14]. Since OA also restored mutant growth and pathogenic development, these data uncover a novel function associated with this molecule that contributes to fungal pathogenic success; the suppression of autophagy.

Oxalic acid (dicarboxylic acid) is a simple, yet remarkably multi-functional, organic acid and non-host selective fungal toxin that is a key component for the pathogenic success of *Sclerotinia*. A2 mutants unexpectedly but consistently, exhibited features associated with an HR response following host inoculation [14]. This is noteworthy as it suggests that the plant can recognize and sense the presence of the mutant and respond defensively in a manner akin to biotrophic resistant responses. Although cell death also occurs following wild type *S. sclerotiorum* challenge none of the HR markers were observed and in fact, there was a pronounced suppression of the plant oxidative burst [13], [14]. This is curious as the oxidative burst has sensibly been considered beneficial to necrotrophs, facilitating disease development [27]. However, we do not believe this is the case in *S. sclerotiorum* for several reasons; (i) when (HR-like) cell death is prevented, the avirulent A2 mutant is pathogenic; (ii) the wild type compatible interaction suppresses the HR-like response and associated markers. If this burst is not suppressed as in the case of the A2 mutant response, resistance is observed. Though we observed an increase in the necrotrophic defense marker PDF1.2 following A2 inoculation, biotrophic defense markers such as callose deposition and the oxidative burst were also evident in the same interaction [14], thus strict lines and definitions of these interactions are blurred. Overall, these responses collectively serve to restrict A2 mutant growth and pathogenic development, however, the molecular details underlying this interaction are incomplete Thus it is apparent, at least with *S. sclerotiorum*, that the fungus/OA suppresses plant defense responses prior to disease development. *S. sclerotiorum* has been considered an aggressive necrotroph. Under suitable environmental conditions, with its battery of weapons including OA and a plethora of cell wall degrading enzymes, it is believed to simply overwhelm the defenseless plant, consistent with its broad host range of essentially all broad leaf plants. In this study and in accord with previous observations [5], [13], [14], we suggest that the fungus does not just overwhelm the host to achieve pathogenic success, but rather co-opts and re-programs plant pathways to generate a suitable environment for pathogen growth and development eventually leading to cell death and a diseased state. Taken together, these results indicate that this plant-pathogen interaction is more complex and subtle than previously thought.

Several recent studies have indicated a role for autophagy in plant defense. Liu et al. (2005) [4] were the first to report a role for autophagy in N-mediated HR-PCD in response to tobacco mosaic virus (TMV). The TMV-tobacco interaction is a key example of a gene-for-gene effector triggered immunity (ETI) recognition model that appears to be mediated by autophagy. Silencing of the autophagy gene *ATG6/BECLIN1*in *Nicotiana benthamiana*, resulted in a reduction of localized (HR) cell death and the unrestricted spreading of cell death emanating from the site of infection. Thus TMV induced plant autophagy is required to constrain pathogen growth and movement suggesting that autophagy is an important component of N mediated plant defense, exhibiting a pro-survival function. A similar phenotype was reported in *ATG6/BECLIN1* silenced *Arabidopsis* plants challenged with *Pseudomonas syringae* carrying the avrRpm1 effector [21]. Thus, autophagy may be mechanistically involved with the HR.

Hofius et. al (2011) [28] reported that autophagy is involved and has a pro-death function in *Arabidopsis* infected with *P. syringae* DC3000 expressing avrRps4. Wild type plants displayed an HR and localized cell death upon challenge with *P. syringae* DC3000; *atg6* mutants on the other hand displayed (partially) reduced HR upon challenge and spread of infection. This cell death phenotype was induced by some, but not all avirulence genes in their corresponding R backgrounds. In these studies, autophagy contributed to resistance and served to limit the spread of HR/PCD, but other components or cell death pathways must also be operative. Consequently, in the TMV/tobacco interaction, autophagy contributes to a pro-survival function whereas Hofius and colleagues concluded a pro-death role of autophagy during the HR, raising a question previously asked in mammalian studies; is autophagy pro-survival or pro-death?

The role of autophagy in response to fungal pathogens has been investigated in several studies; however, the mechanistic details controlling autophagy to modulate fungal challenge are incomplete. Autophagy deficient (T-DNA knockout) *Arabidopsis* plants were more resistant to the biotrophic fungus *Golovinomyces cichoracearum* in a salicylic acid dependent manner [29]. Marked resistance to the virulent biotrophic pathogen *Pseudomonas syringae* pv. *tomato* was also observed in *atg* mutant plants [30]. These studies suggest a negative effect of plant autophagy in resistance responses toward biotrophic pathogens and are in contrast to N mediated resistance to TMV, which required functional autophagic machinery for an effective defense response [4]. Thus, the contribution of autophagy to plant defense may be different, even within the same pathogen (biotrophic) life style.

In contrast to biotrophs, which require living cells for nutrition, growth and pathogenic development, necrotophic pathogens feed on dead or dying cells and typically promote cell death during the early stages of infection. Thus, host cell death via autophagy might support invasion and colonization by necrotrophic pathogens. As noted above, it has been suggested that necrotrophic fungi exploit the plant HR for this reason [27]. However, *Arabidopsis* mutants deficient in autophagy are more susceptible to the necrotrophic pathogens, *Botrytis cinerea* and *Alternaria brassicicola* [31]. The susceptibility of *atg* mutant plants to *A. brassicola* was further confirmed by Lenz et al (2011) [30] and was accompanied by ROS production and enhanced hyphal growth.

Given the diverse roles of autophagy and apoptosis in innate immunity, it is not surprising that microbial pathogens have devised strategies to exploit PCD to gain the upper hand and circumvent host defense. Intracellular bacteria and viruses are well documented to subvert the autophagic machinery. *Coxiella burndeti* and *Legionella pneumophila* actually benefit from autophagy as autophagic structures provide a niche for bacterial replication [32]. Autophagy also plays a key protective role in mediating immune responses against several bacterial pathogens. *Salmonella typhimurium*, *Mycobacterium tuberculosis* and *Streptococcus* have all been reported to be trapped in autophagosomal structures in resistant mammalian cell lines. In autophagy mutant backgrounds, these structures are absent and the bacteria multiply [33]. An interesting infection strategy is illustrated during *Shigella* infection of epithelial cells. The bacterium damages host mitochondria and causes oxidative stress which eventually kills host cells. During the early stages of infection, *Shigella* effectively counteracts the strong host cytotoxic response by inducing a pro-survival pathway mediated by NF-kB [34]. NF-kB is an important transcription factor that triggers cytoprotective anti-apoptotic genes (eg *Bcl-2*) that prevent the death of *Shigella* infected cells. This prolonged survival of host cells allow the bacteria time to replicate and spread [35].

We have also suggested that host survival during the early stages of infection is of benefit to the invading microbe by affording *S. sclerotiorum* valuable time for unimpeded establishment of infection. During the early stages of colonization, OA triggers a reducing environment that is correlated with the ability of OA to dampen the plant oxidative stress response; plant cells remain viable and do not appear to recognize the presence of the fungus allowing time for establishment. Once this occurs, it is too late for the plant to effectively respond and fungal induced apoptotic PCD and disease ensues.

Intriguingly, during these early stages of fungal colonization, plant cells are viable as determined by trypan blue staining, while the fungus grows in the apoplast. This is notable as *S. sclerotiorum* has been exclusively associated with classic necrotrophic pathogenesis and surface growth. This raises the question as to whether *S. sclerotiorum*, an aggressive prototypical necrotrophic pathogen armed with a battery of cell wall degrading enzymes and secondary metabolites, including OA, is truly a necrotroph? We suggest that this fungus is more accurately a hemibiotroph, a significant shift in how this pathogen and pathogenesis is viewed. During the initiation of pathogenesis, the wild type fungus does not kill cells; there is no evidence for oxidative stress and fungal growth is observed in living plant tissue.

How would *S. sclerotiorum* initiate biotrophy? We have shown [13], [14] that wild type/OA but not OA mutants, dampen the oxidative burst and maintain biotrophy before a switch to PCD. Experiments are in progress to define this biotrophic phase of growth. This biotrophic phase need not to be long or extensive. The biotrophic phase of the hemibiotroph *Colletotrichum higginsianum*, for example, is restricted to one host cell and is followed by a complete transition to necrotrophy [36]. This raises the interesting question of whether A2's growth on autophagy defective plants is entirely biotrophic. We were unable to detect any evidence for biotrophy; in addition host tissue treated with chemical autophagy inhibitors positively stained with trypan blue following inoculation of A2 (Fig. 5C). Though OA is a key pathogenicity factor, we know it is not the only factor conditioning pathogenicity, thus despite A2's inability to overcome the autophagic defense response mounted by wild type plants, this strain presumably harbors additional factors in its arsenal that contribute to disease. This is consistent with the fact that OA mutant challenge of autophagy deficient plants is intermediate in virulence compared to the wild type fungal strain.

It is tempting to speculate that fungal effectors contribute to this biotrophy/necrotrophy life style in *S. sclerotiorum*. Necrotrophic fungal effectors have been reported in the wheat pathogen *Stagonospora nodorum* that produces the host-selective toxin/effector SnTox1 [37]. This toxin induces susceptibility in wheat and this interaction has been termed “effector-triggered susceptibility” in contrast to the much discussed “effector triggered immunity” (ETI) [38]. *S. sclerotiorum* has not been reported to harbor effectors. However, we recently identified an effector-like protein in *Sclerotinia* (SsCm1) that has strong similarity (structural and functional) to the *Ustilago maydis* effector Cmu1, encoding a secreted chorismate mutase. It has been proposed that Cmu1 serves to maintain biotrophy during fungal establishment in maize [39] and we speculate that this might be the case with *Sclerotinia*.

The lines of distinction between autophagy and apoptosis are often blurred as they both regulate cell death. Moreover, the same gene product can modulate both processes. For example, Bcl-2 (an anti-apoptotic oncogene) was shown to bind to and inhibit Beclin1, resulting in the prevention of autophagy [40]. Bcl-2 can therefore function as an anti-autophagy protein along with its well known role as an anti-apoptotic protein. Atg12 promotes autophagy but can bind and inhibit Bcl-2 promoting apoptosis [41]. Our initial data indicates that the wild type fungus/OA inhibits autophagy and apoptosis is observed. Whether apoptosis occurs by default in the absence of autophagy or actively suppresses/overrides autophagy is not clear. The molecular details as to how OA regulates these PCD pathways are also incomplete.

**Figure 7:**
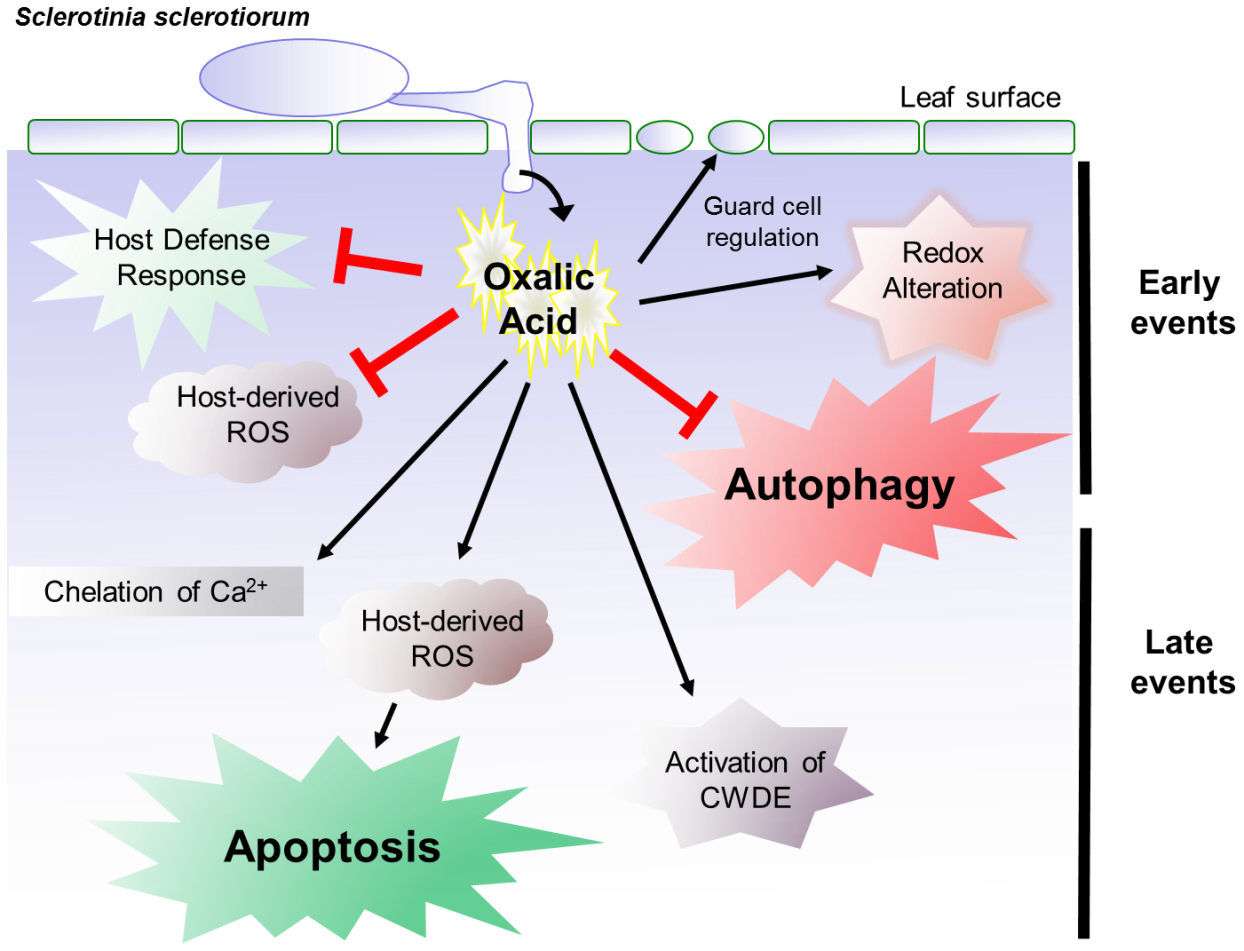
Oxalic acid is multifunctional. OA is a pathogenicity determinant in *Sclerotinia* that has a number of functions that facilitate fungal pathogenicity. OA inhibits plant defense responses (eg callose deposition) and modulates the host redox environment by blocking the host oxidative burst and creating reducing environment. OA also suppresses autophagy. At later stages, OA accumulation lowers the pH, activates cell wall degrading enzymes and a MAP kinase required for pathogenic sclerotial development. This process culminates in OA induced ROS leading to elicitation of apoptotic cell death and disease. (For further details see Dickman, 2007; Williams et al., 2011).

Figure 7 summarizes our findings. The wild type compatible pathogen (OA^+^) triggers a reducing host cellular environment during the initial stages of pathogen establishment. We suggest that this reducing activity serves to dampen the oxidative burst and plant defense is suppressed in a manner akin to (hemi)-biotrophic pathogens. The fungus transitions into necrotrophy and a fungal controlled (apoptotic-like) compatibility induced cell death and susceptibility occurs. In the A2 strain which is non-pathogenic; plant defense responses are observed; plant controlled (autophagous) cell death is triggered leading to incompatibility induced cell death and resistance. Although this work has focused on cell death mechanisms in the host, a recent report has suggested that the host can also induce PCD in the fungus [42].

There are an increasing number of examples of plant-pathogen interaction strategies that result in pathogen manipulation and subversion of host metabolic cell death pathways to facilitate infection. It is evident that the control of cell death is crucial to the outcome of many plant-fungal interactions. We propose that it is not cell death per se that is key to the outcome of these interactions, but rather the means by which cell death occurs that is decisive.

## Materials and Methods

### Plant and fungal materials

*S. sclerotiorum* wild type isolate 1980 and the oxalate-deficient mutants derived from this strain, were maintained as previously described [14]. Plants were grown from seed and maintained under greenhouse conditions. *Arabidopsis* Col-0 and corresponding T-DNA knock-outs were acquired from the Arabidopsis Stock Center (www.arabidopsis.org). Plants expressing the nematode *ced-9* were generated as described [15]. Pathogenicity assays were conducted as previously described [14].

### Electron microscopy

Leaf segments were fixed for 5 hours at 20°C in 3% glutaraldehyde and 2% formaldehyde in 0.1M Na cacodylate buffer then were processed for transmission electron microscopy according to standard protocols. Briefly, the leaves were post-fixed in 1% osmium tetroxide in 0.1M sodium cacodylate buffer for 1 hour, dehydrated in a graded alcohol series, and embedded in Spurr's epoxy resin (Electron Microscopy Sciences, Hatfield, PA). Semi-thin sections (400 nm thick) were cut with a Leica EM UC6 microtome, transferred to glass slides and attached by heating, then stained with Epoxy Tissue Stain (Electron Microscopy Sciences, Hatfield, PA). Ultrathin sections were cut with a Leica EM UC6 microtome, and post-stained with uranyl acetate and lead citrate. Sections were examined with a Phillips Morgagni 268 (FEI company, Hillsboro, OR) transmission electron microscope at an accelerating voltage of 80 kV. Digital images were recorded with a MegaViewIII digital camera operated with iTEM software (Olympus Soft Imaging Systems, Germany). The Adobe Photoshop CS4 digital photography editing program was used for additional processing.

### ROS detection assay

In situ O2− was detected by nitroblue tetrazolium (NBT; Sigma-Aldrich, St. Louis, MO) staining, (Kim et al., 2008; Williams et al., 2011). Briefly, two days post-inoculation fully expanded Arabidopsis leaves were infiltrated with a 0.5 mg/ml of NBT solution (10 mM potassium phosphate buffer, pH 7.5) for 2 h in the dark at room temperature, chlorophyll was removed by incubation in 70% ethanol and samples mounted in 50% glycerol. All samples were visualized using an Olympus SZx10 stereoscope (Olympus systems, Germany).

### Trypan blue staining and viability

Twenty-four hours post-infection, Sclerotinia challenged leaves and onion epidermal layers were stained with 0.05% Trypan blue for 45 min at 25°C and washed twice with PBS. Plasmolysis was induced by incubating the onion epidermal layer in a 1M sucrose solution for 1 h. All samples were observed using an Olympus SZ610 microscope. Images were collected using Olympus DP controller (Olympus Systems, Germany) and processed using the Olympus FLUOVIEW software, version FV10-ASW 2.0.

### Real-time PCR

Total RNA was isolated using the plant RNeasy kit (Qiagen, Hilden, Germany). Samples were treated with RNase-free Dnase (Qiagen) to remove DNA contamination according to the manufacturer's instructions. One microgram of total RNA was reverse transcribed by MLV-reverse transcriptase (Life Technologies, NY, USA) using an oligo (dT) (100 ρmole) primer. Quantitative PCR was carried out with an ABI 7900HT cycler, using SYBR Green PCR master mix (Applied Biosystems, USA) as described by the manufacturer, using the following cycling parameters: 50°C, 95°C, (96°C for 6 s, 60°C for 1 min) for 40 cycles. Gene-specific primers were designed using the Primer3 bioinformatic software (MIT). Arabidopsis UBQ10 was used as a loading control.

### MDC and lysotracker staining

Detached tomato leaves were agar plug inoculated with wild type and A2 fungal strains. Twenty four hours post inoculation leaves were stained with a 100 µM final concentration of Mono-Dansyl Cadaverine MDC (Sigma, USA) in PBS for 30 min. Leaves were then washed twice with PBS to remove excess MDC. LysoTracker (Invitrogen, USA) was diluted from a 1 mM stock to a 100 nM working solution in ½ MS. Leaves were incubated for one hour and then washed twice with ½ MS before visualization. Fluorescence was visualized using an Olympus IX81 inverted fluorescence confocal microscope (Olympus systems, Germany). The excitation and emission wavelengths of 335 nm and 508 nm, respectively, were used for MDC, Lysotracker excitation and emission wavelengths were 465 nm and 535 nm, respectively. All images were collected using Olympus DP controller (Olympus Systems, Germany). Images were processed using the Olympus FLUOVIEW software, version FV10-ASW 2.0.

### Cell death detection

DNA ladders were detected by extracting total DNA from homogenized leaf discs 48 hrs following OA treatment. Samples were incubated for 30 min at room temperature in DNA extraction buffer (0.1 M glycine, 50 mM NaCl, 10 mM EDTA, 2% SDS, and 1% sodium lauryl sarcosine) and mixed with an equal volume of Tris-saturated phenol. The mixture was centrifuged for 15 min at 10,000× g. The supernatant was treated with chloroform/isoamyl alcohol (24∶1, vol/vol). After centrifugation as above, DNA was precipitated with a twofold volume of 100% ethanol, washed with 70% ethanol, and dissolved in Tris-EDTA buffer containing RNase A (40 µg/ml). DNA was recovered after phenol extraction and ethanol precipitation. DNA samples were separated on a 1.5% agarose gel in 0.5× Tris-borate-EDTA (1×89 mM Tris, 89 mM H3BO3, and 2 mM EDTA), stained with ethidium bromide, and visualized under UV light.

TUNEL positive nuclei were detected using the DeadEnd Fluorometric TUNEL System (Promega Corporation, Madison, WI) following the manufacturer's recommendations.

## Supporting Information

Figure S1 ***S. sclerotiorum*** **grows biotrophically in onion epidermal cells.** Wild type *S. sclerotiorum* was inoculated onto onion epidermal cells placed on a microscope slide. 24 hours post inoculation; tissue was stained with Trypan blue (A) to reveal fungal hyphae and determine viability of onion cells. Thick invasive hypha is shown growing within living tissue. Sucrose induced plasmolysis was used to further verify the viability of these cells (B). Images were collected using an Olympus DP 70 camera and processed with Olympus DP Controller software, version 2.2.1.227.
(JPG)

Figure S2 **LysoTracker staining of A2 and wild type infected tomato leaves.** *S. sclerotiorum* wild type and A2 strains were inoculated onto tomato leaves via agar plugs. 24 hours post inoculation; both inoculated and non-inoculated leaves were stained with 75 nM final concentration of LysoTracker Green (Invitrogen) in PBS for 2 hours. Fluorescence was visualized using an Olympus stereoscope SZX 10 (Olympus systems, Germany), with an excitation wavelength of 504 nm and an emission wavelength of 511 nm. Scale bar = 100 µm. Images were collected using an Olympus DP 70 camera and processed with Olympus DP Controller software, version 2.2.1.227.
(JPG)

Figure S3 ***Arabidopsis*** **autophagy mutants are sensitive to the OA deficient** ***sod1*** **mutant.** Agar plugs containing actively growing cultures of wild type and the *Δsod1* mutant were inoculated onto *Arabidopsis* Col-0 and a series of autophagy mutant plants. These mutants showed enhanced susceptibility to *Δsod1* compared to Col-0 plants. Infection was monitored over time, and all images were recorded 60 hours post inoculation.
(JPG)

Figure S4 **Mono Dansyl Cadavirine (MDC) staining of A2 infected leaf tissue with and without OA pretreatment.** *S. sclerotiorum* wild type and A2 strains were inoculated onto tomato leaves (top panel) or tomato leaves pre-infiltrated with KOA pH 7 (bottom panel) using agar plugs. 24 hours post inoculation; leaves were stained with 100 µM final concentration of MDC (Sigma) in PBS for 30 min. Fluorescence was visualized using an Olympus IX81 inverted fluorescence confocal microscope (Olympus systems, Germany), with an excitation wavelength of 335 nm and an emission wavelength of 508 nm. Images were collected using an Olympus DP 70 camera and processed with Olympus DP Controller software, version 2.2.1.227. Scale bar = 10 µm.
(JPG)

Figure S5 **Expression analysis of autophagy and defense related genes in A2 and wild type *S. sclerotiorum* infections.** (A) Quantitative RT-PCR was used to evaluate the transcript levels of respective autophagy genes in wild-type Col-0 plants upon challenge with A2 (gray bars) and wild type (black bars) S. sclerotiorum. (B) Transcript accumulation of defense-related genes PR1, PDF1.2, and PR5 in Col-0 leaves following inoculation with A2 (gray bars) and wild type (black bars) S. sclerotiorum. UBQ10 gene expression served as the loading control. * >2 fold change, ** >5 fold change.
(JPG)

Figure S6 **Non-inoculated tomato leaves stained with Monodansyl Cadavirine (MDC).** Tomato leaves were stained with 100 µM final concentration of MDC (Sigma) in PBS for 30 min. Fluorescence was visualized using an Olympus IX81 inverted fluorescence confocal microscope (Olympus systems, Germany), with an excitation wavelength of 335 nm and an emission wavelength of 508 nm. Images were collected using an Olympus DP 70 camera and processed with Olympus DP Controller software, version 2.2.1.227. Scale bar = 10 µm.
(JPG)
