# Supplementary figures and images for: Cell Death Control: The Interplay of Apoptosis and Autophagy in the Pathogenicity of Sclerotinia sclerotiorum
Source: PLoS Pathog. 2013 Apr 11;9(4):e1003287. doi: 10.1371/journal.ppat.1003287 (PMC3623803; doi:10.1371/journal.ppat.1003287)

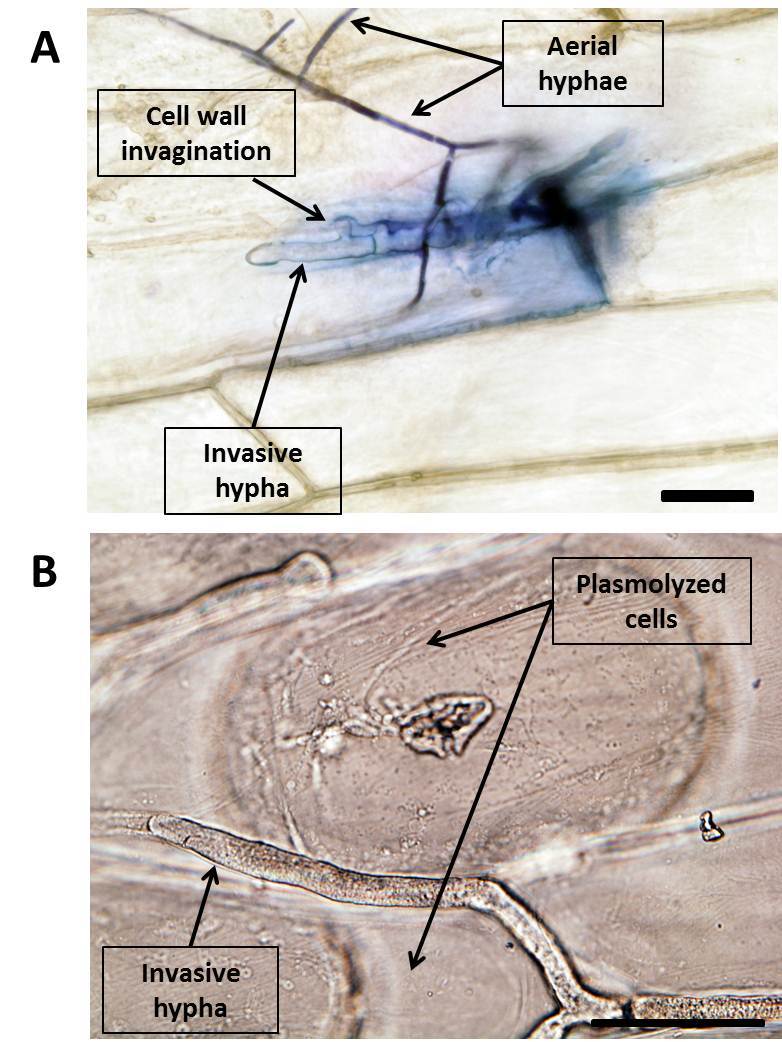

Supplement: Figure S1 — S. sclerotiorum grows biotrophically in onion epidermal cells. Wild type S. sclerotiorum was inoculated onto onion epidermal cells placed on a microscope slide. 24 hours post inoculation; tissue was stained with Trypan blue (A) to reveal fungal hyphae and determine viability of onion cells. Thick invasive hypha is shown growing within living tissue. Sucrose induced plasmolysis was used to further verify the viability of these cells (B). Images were collected using an Olympus DP 70 camera and processed with Olympus DP Controller software, version 2.2.1.227. (JPG) [file ppat.1003287.s001.jpg]

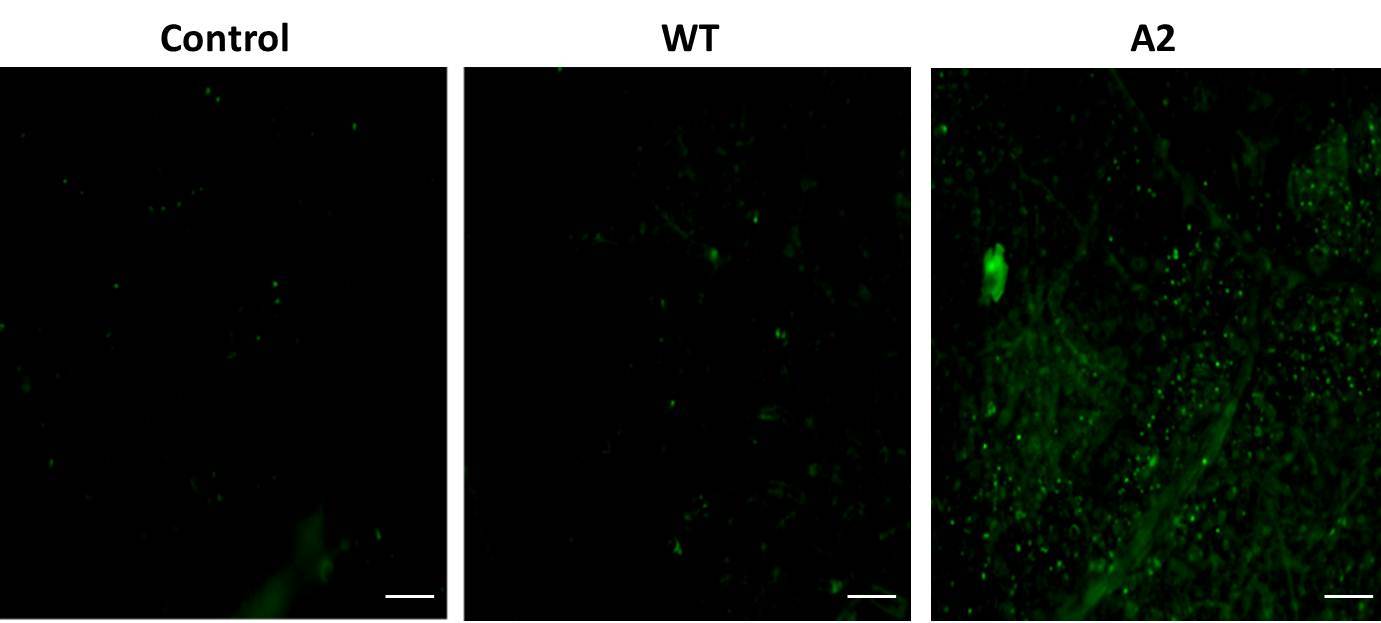

Supplement: Figure S2 — LysoTracker staining of A2 and wild type infected tomato leaves. S. sclerotiorum wild type and A2 strains were inoculated onto tomato leaves via agar plugs. 24 hours post inoculation; both inoculated and non-inoculated leaves were stained with 75 nM final concentration of LysoTracker Green (Invitrogen) in PBS for 2 hours. Fluorescence was visualized using an Olympus stereoscope SZX 10 (Olympus systems, Germany), with an excitation wavelength of 504 nm and an emission wavelength of 511 nm. Scale bar = 100 µm. Images were collected using an Olympus DP 70 camera and processed with Olympus DP Controller software, version 2.2.1.227. (JPG) [file ppat.1003287.s002.jpg]

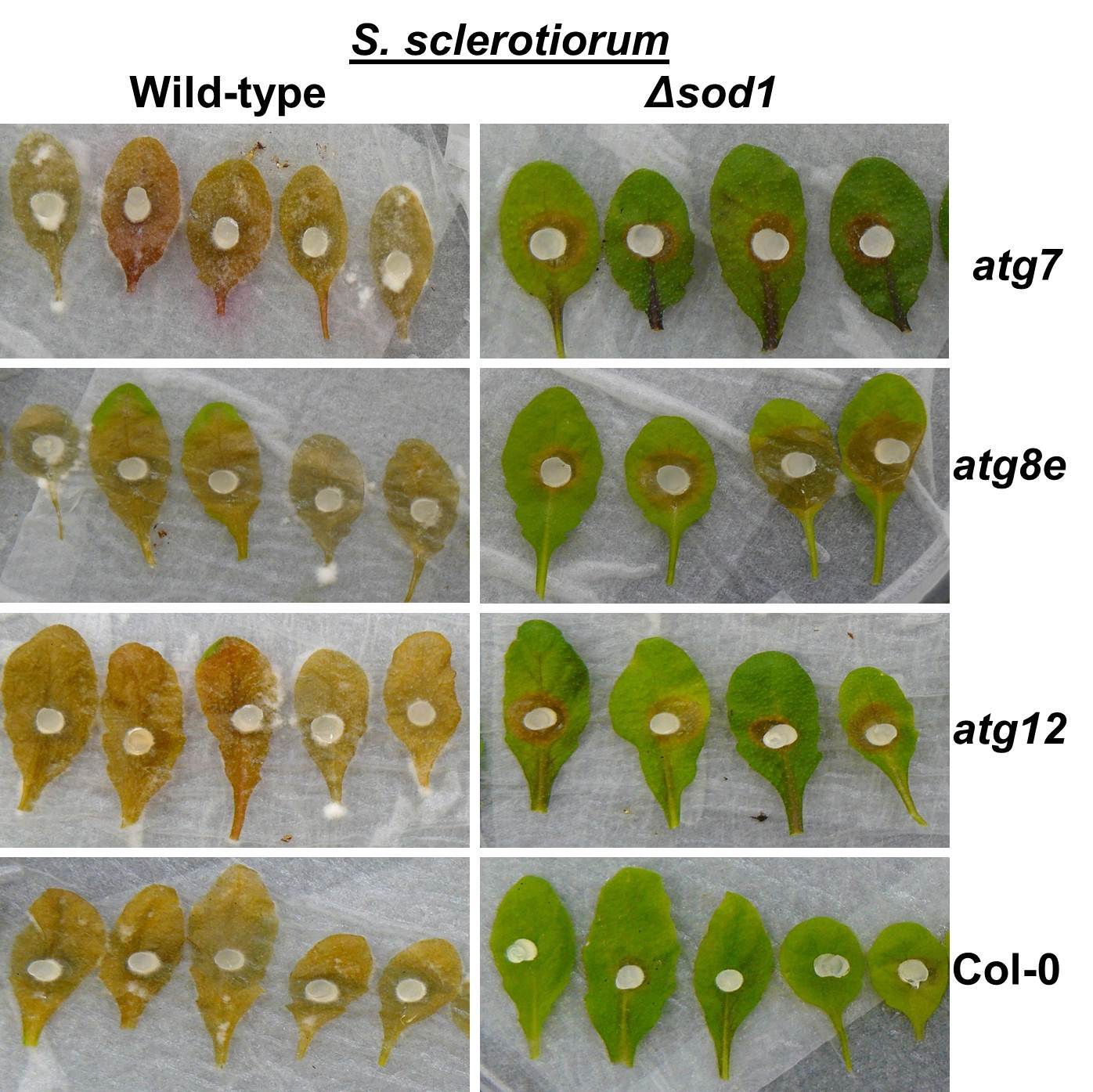

Supplement: Figure S3 — Arabidopsis autophagy mutants are sensitive to the OA deficient sod1 mutant. Agar plugs containing actively growing cultures of wild type and the Δsod1 mutant were inoculated onto Arabidopsis Col-0 and a series of autophagy mutant plants. These mutants showed enhanced susceptibility to Δsod1 compared to Col-0 plants. Infection was monitored over time, and all images were recorded 60 hours post inoculation. (JPG) [file ppat.1003287.s003.jpg]

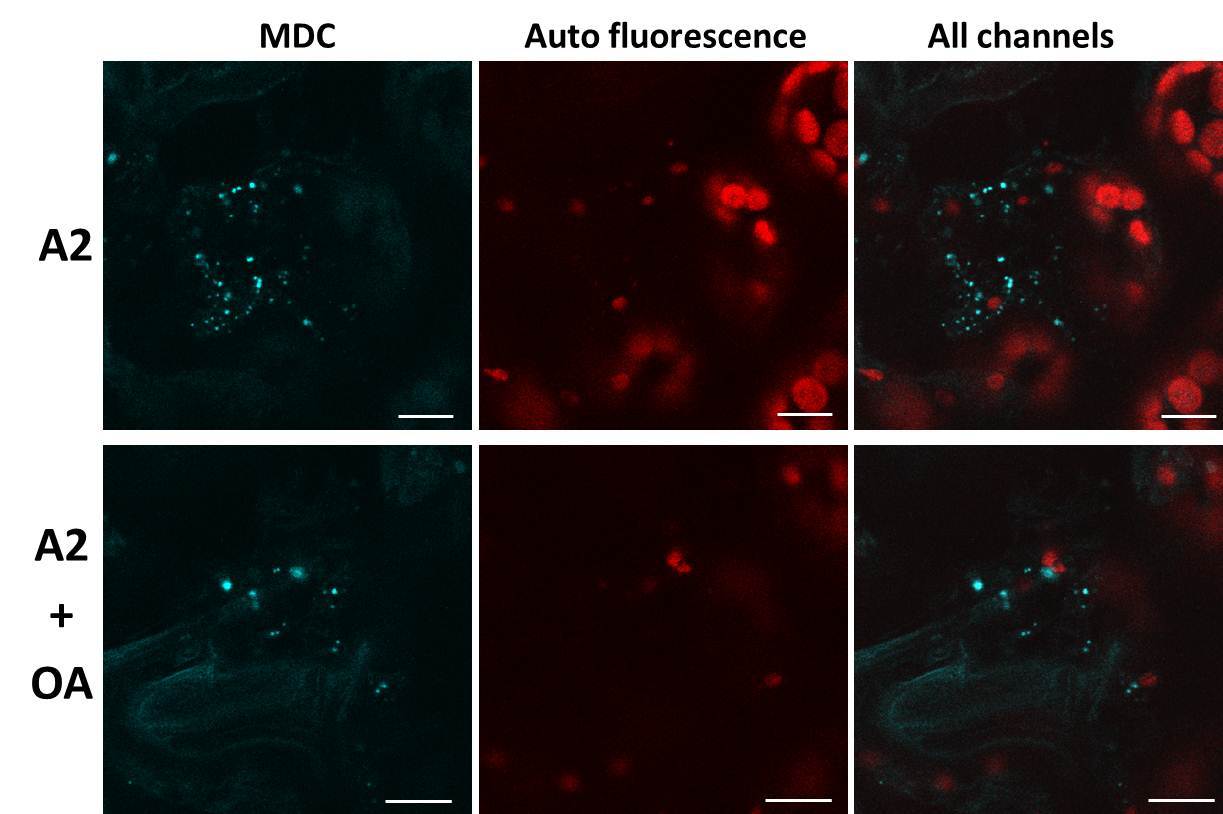

Supplement: Figure S4 — Mono Dansyl Cadavirine (MDC) staining of A2 infected leaf tissue with and without OA pretreatment. S. sclerotiorum wild type and A2 strains were inoculated onto tomato leaves (top panel) or tomato leaves pre-infiltrated with KOA pH 7 (bottom panel) using agar plugs. 24 hours post inoculation; leaves were stained with 100 µM final concentration of MDC (Sigma) in PBS for 30 min. Fluorescence was visualized using an Olympus IX81 inverted fluorescence confocal microscope (Olympus systems, Germany), with an excitation wavelength of 335 nm and an emission wavelength of 508 nm. Images were collected using an Olympus DP 70 camera and processed with Olympus DP Controller software, version 2.2.1.227. Scale bar = 10 µm. (JPG) [file ppat.1003287.s004.jpg]

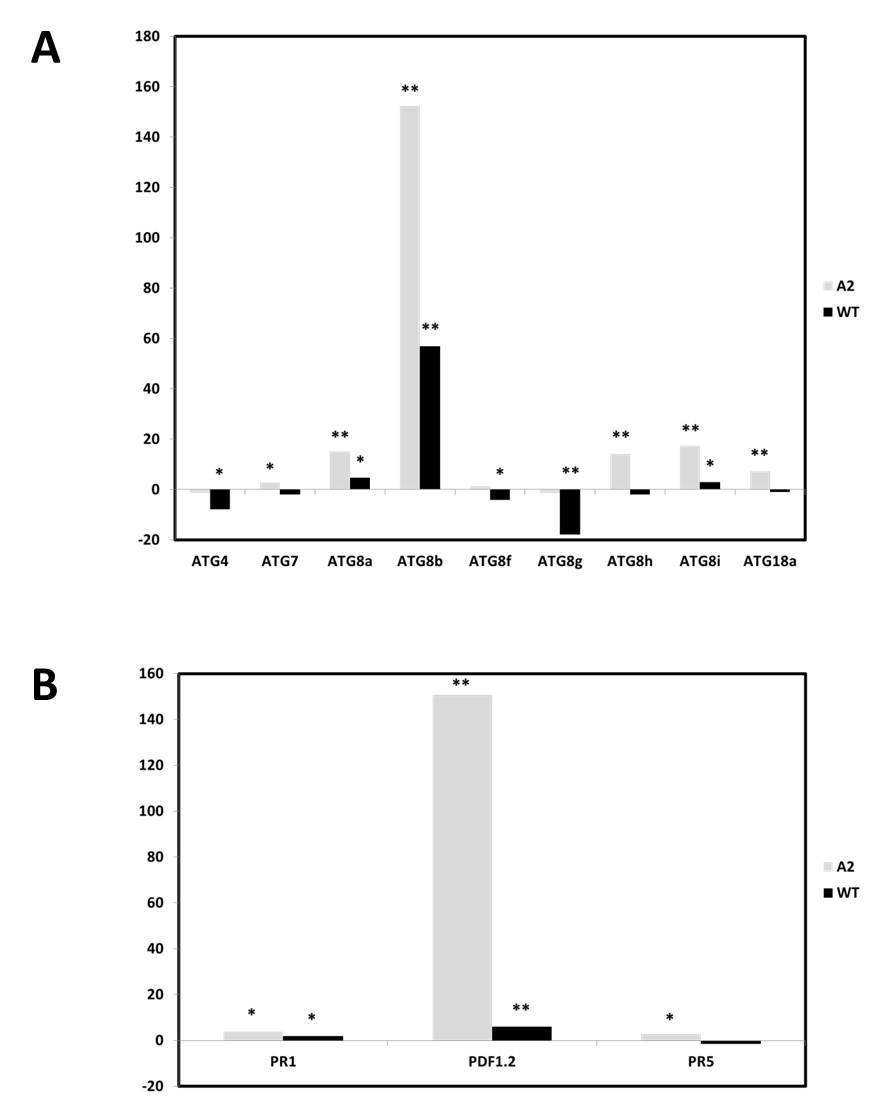

Supplement: Figure S5 — Expression analysis of autophagy and defense related genes in A2 and wild type S. sclerotiorum infections. (A) Quantitative RT-PCR was used to evaluate the transcript levels of respective autophagy genes in wild-type Col-0 plants upon challenge with A2 (gray bars) and wild type (black bars) S. sclerotiorum. (B) Transcript accumulation of defense-related genes PR1, PDF1.2, and PR5 in Col-0 leaves following inoculation with A2 (gray bars) and wild type (black bars) S. sclerotiorum. UBQ10 gene expression served as the loading control. * >2 fold change, ** >5 fold change. (JPG) [file ppat.1003287.s005.jpg]

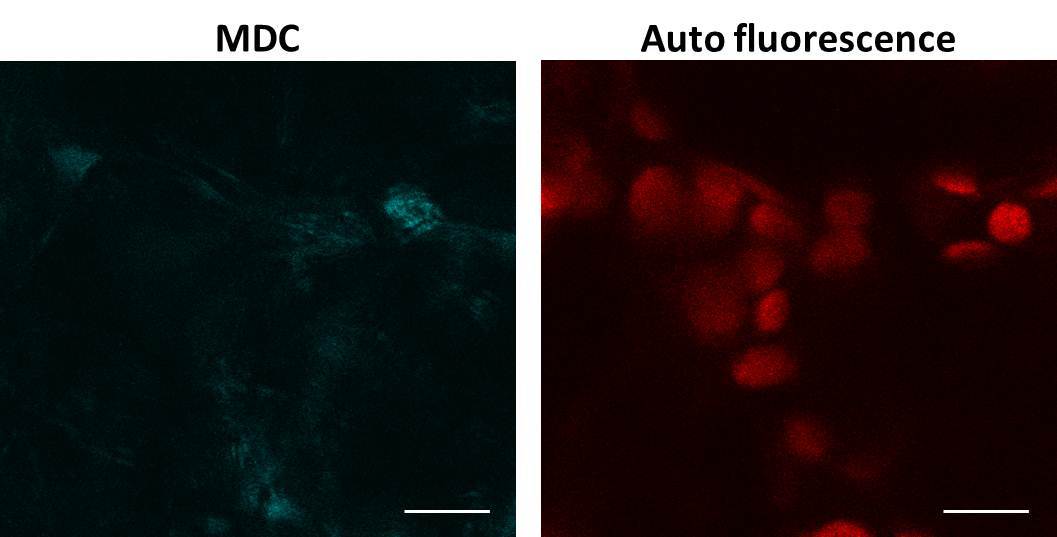

Supplement: Figure S6 — Non-inoculated tomato leaves stained with Monodansyl Cadavirine (MDC). Tomato leaves were stained with 100 µM final concentration of MDC (Sigma) in PBS for 30 min. Fluorescence was visualized using an Olympus IX81 inverted fluorescence confocal microscope (Olympus systems, Germany), with an excitation wavelength of 335 nm and an emission wavelength of 508 nm. Images were collected using an Olympus DP 70 camera and processed with Olympus DP Controller software, version 2.2.1.227. Scale bar = 10 µm. (JPG) [file ppat.1003287.s006.jpg]
